# Introducing entrustable professional activities for postgraduate medical training in Switzerland

**DOI:** 10.3205/zma001715

**Published:** 2024-11-15

**Authors:** Severin Pinilla, Werner Bauer, Jan Breckwoldt, Christoph S. Burkhart, Eva K. Hennel, Adrian P. Marty, Urs von Wartburg, Monika Brodmann Maeder, Sören Huwendiek

**Affiliations:** 1 University of Bern, Institute for Medical Education, Department for Assessment and Evaluation, Bern, Switzerland; 2 University of Bern, University Hospital of Old Age Psychiatry and Psychotherapy, Bern, Switzerland; 3 Swiss Institute of Medical Education (SIME), EPA-Commission, Bern, Switzerland; 4 University Hospital Zurich, Institute of Anesthesiology, Zurich, Switzerland; 5 Kantonsspital Graubünden, Department of Anesthesiology, Graubünden, Switzerland; 6 University Hospital Balgrist, Zurich, Switzerland; 7 Swiss Institute of Medical Education (SIME), Bern, Switzerland

**Keywords:** competency-based medical education, entrustable professional activities, faculty development, change management

## Abstract

**Introduction:**

Graduate medical education is being reformed in many countries, with a focus on the principles of competency-based medical education (CBME). A main novel aspect in this context is the implementation of entrustable professional activities (EPAs). The introduction of EPAs aims to better align training curricula with clinical practice, provide individualized supervision, and enhance the quality of feedback.

**Project description:**

This project report presents the development of a national strategy and the initial results of implementing entrustable professional activities in the Swiss context.

**Results:**

Affiliated with the Swiss Institute of Medical Education (SIME), an EPA-Commission was established with the mandate to develop a strategy and provide guidance to medical specialty societies. To date, 28 out of 45 specialty societies have sought advice from the EPA-Commission and have begun developing EPAs. Concurrently, the Commission has expanded the national faculty development courses, adapted the content, started offering multilingual courses, and has published a series of articles on CBME and EPAs. Selected pilot hospitals are now planning to implement EPA-based graduate medical education curricula. Additionally, the introduction of a nationwide electronic solution (app) for assessing EPAs is planned.

**Conclusion:**

The introduction of EPAs in graduate medical education is a multilayered project. In addition to medical education aspects, various social, organizational, and professional-political factors are crucial for the transformation processes. In the Swiss context, such a reform has been successfully initiated. Continuous evaluations of the ongoing projects will provide further insights for competency-based graduate medical education reforms.

## Zusammenfassung

**Einleitung:**

Die ärztliche Weiterbildung wird in zahlreichen Ländern reformiert. Im Zentrum stehen dabei die Prinzipien der kompetenzbasierten Aus- und Weiterbildung (Competency-based Medical Education, CBME). Eine wesentliche Neuerung in den letzten Jahren ist hierbei die Arbeit mit Entrustable Professional Activities (EPA). Deren Einführung zielt wesentlich auf eine bessere Passung von Weiterbildungscurricula und klinischer Arbeit, individualisierter Supervision und Verbesserung der Feedbackqualität ab.

**Projektbeschreibung:**

Im vorliegenden Projektbericht werden eine nationale Strategieentwicklung und erste Ergebnisse der Entwicklung von Entrustable Professional Activities im schweizerischen Kontext dargestellt.

**Ergebnisse:**

Angegliedert an das schweizerische Institut für ärztliche Weiter- und Fortbildung (SIWF) wurde eine EPA-Kommission mit dem Auftrag einer Strategieentwicklung und Beratung der Fachgesellschaften eingerichtet. Insgesamt 28 von 45 Fachgesellschaften haben bisher die Beratung durch die EPA-Kommission für die EPA-Entwicklung in Anspruch genommen und mit der EPA-Entwicklung begonnen. Begleitend wurde durch die Kommission das nationale Fakultätsentwicklungsangebot inhaltlich und mehrsprachig weiterentwickelt und eine Artikelserie zu CBME und EPAs publiziert. Ausgewählte Pilotspitäler planen nun EPA-basierte Implementierungen entsprechender Weiterbildungscurricula. Zudem ist die Einführung einer landesweiten elektronischen Lösung (APP) zur Erfassung der EPAs vorgesehen.

**Schlussfolgerung:**

Die Einführung von EPAs in der ärztlichen Weiterbildung ist ein komplexes Projekt. Dabei spielen verschiedene gesellschaftliche, organisatorische und berufspolitische Rahmenbedingungen, neben den medizindidaktischen, eine entscheidende Rolle für Umgestaltungsprozesse. Im schweizerischen Kontext konnte eine solche Reform erfolgreich gestartet werden. Kontinuierliche Evaluationen der laufenden Projekte werden weitere Erkenntnisse für kompetenzbasierte Weiterbildungsreformen liefern.

## Introduction

Graduate medical education is being reformed in many countries, with a focus on the principles of competency-based medical education. Over the past 15 years, numerous articles on empirical studies and educational project reports in this context have been published in the international medical education literature [1], [2], [3], [4], [5], [6], [7], [8], [9]. The earlier articles [1], [3], [4] in the sense of a critical assessment of the status quo and the call to reform graduate medical education in a patient-oriented, competency-based and learner-centered way, the later articles [2], [5], [6], [7], [8], [9] in the sense of a reflection on what has been achieved so far and the future reform strategy.

In English-speaking countries (especially Canada, the USA and Australia), competency-based medical education, including the introduction of entrustable professional activities in undergraduate and graduate medical education, is already well advanced in some cases [2]. In Switzerland and Germany, the focus was initially on competency-based undergraduate medical education [10], [11], [12]. Only recently, initiatives have been started to reform graduate medical education in these countries as well [13], [14]. In the Swiss context, following the introduction of entrustable professional activities (EPAs) in undergraduate medical education [10], a national strategy has been developed to transform graduate medical education by introducing EPAs based on competencies [13]. 

Graduate medical education reforms in most European countries partly face different challenges compared to Anglo-American regions due to health system and professional policy context factors. For example, one major difference is the lack of centralized residency allocation for standardized residency training curricula. The primary responsibility for organizing graduate medical training rotations typically lies with the residents themselves. This is a significant difference compared to the standardized residency programs in the USA. The perception of residents in Switzerland and Germany tends to be more dominated by their role as healthcare workers and only secondarily as learners. Those responsible for graduate medical education must therefore typically subordinate aspects of residency training to those of patient care. The clinical rotations and their duration in graduate medical education therefore often do not follow a fixed curriculum but are subject to organizational needs (where is someone needed versus what should the resident learn).

At the healthcare system level, it should also be noted that not all countries have a national institute with a medical education mandate to coordinate a national residency reform. Typically, numerous different institutions at state or cantonal level or departments at universities shape undergraduate and graduate medical education activities. For a systematic reform of graduate medical education, professional policy institutions (e.g. regional medical chambers) and departments of the ministries of health must be involved in addition to the medical societies.

With this project report, the authors would like to critically reflect on Swiss efforts and experiences with a national competency-based graduate medical education reform, focusing on the introduction of EPAs for different specialties and make them useful for ongoing projects.

## Project description

### Context of Swiss graduate medical education

An overview of all graduate medical education programs can be found on the homepage of the Swiss Institute of Medical Education (SIME) [15]. The respective specialist societies are responsible for defining the content of the graduate medical education programs in the Swiss context. The certification and re-certification of the corresponding residency programs is carried out by the SIME together with the respective specialist societies. The residency program directors (usually the clinic directors) are responsible for the implementation and realization of the programs. As board certification requirement, different types of teaching hospital and rotations (academic medical centers versus primary care hospitals, as well as outpatient and inpatient settings) must be completed. 

Residents themselves are responsible for the corresponding applications and organization of their rotations between teaching hospitals (translator note: the residents apply for individual clinical employment, not residency programs). Residents also self-initiate signing up for the written and oral board examinations that are organized by the medical societies. A residency rotation certificate is issued after each period of graduate medical training or at least once a year. This is documented in a central electronic logbook and checked by the SIME at the end of the residency. 

As well as certifying residency training hospitals and checking entries in the e-logbook, the SIME is also responsible for ensuring the quality of graduate medical education through annual evaluations of the training centers by residents and in-person site visits to the training centers (visitations).

At national level, the Federal Office of Public Health [FOPH, German: Bundesamt für Gesundheit (BAG)] is responsible for implementing the legal framework for graduate medical education for all healthcare professions in Switzerland [15]. In addition, the recognition of graduate medical training qualifications via bilateral EU agreements is subject to the corresponding EU directives.

### Strategy development of the Swiss Institute for Medical Education

In preparatory SIME workshops led by the SIME presidents (Dr. med. Werner Bauer 2010-2021, PD Dr. med. et MME Monika Brodmann-Mäder since 2021), the strategic direction for Swiss competency-based medical education for ten years has been developed since 2021. In addition to working sessions with invited international experts (including Prof. Olle ten Cate, Prof. Jason Frank), a longer-term international advisory structure (“advisory board”) has also been established. The initially developed strategy for competency-based medical education was presented and discussed at numerous specialist conferences and coordination meetings with the FOPH. An information platform was set up on the SIME website to provide documents for the medical societies and their respective EPA working groups [15].

The content developed was made available as part of a series of publications [13], [15], [16], [17], [18], [19], [20], [21], [22], [23], [24] for the Swiss Medical Journal (bilingual, German and French). In addition, medical education research projects were designed to explore the development and implementation of EPAs in the Swiss graduate medical education context.

A core working group (EPA Commission) was established and organizationally assigned to the SIME. In its composition, attention was paid to broad clinical and medical education experience. The commission was supplemented by a research unit and administrative support.

### Faculty development

The existing faculty development program in cooperation with the Royal College of Physicians of London was revised to reflect the new competency-based orientation [25].

## Results

### EPA Commission

A bimonthly meeting frequency was implemented for the EPA Commission (as a hybrid format of online and face-to-face participation). At the meetings, the overarching strategic direction was discussed, and the respective specialist society consultations were deliberated and agreed upon. The overarching goal was defined as the improvement of graduate medical education in Switzerland through the introduction of competency-based graduate medical education incorporating EPAs. The SIME decided that all future revisions of training programs must adhere to this premise. Additionally, a working group was established to decide on a digital solution for the documentation of workplace-based assessments. This group developed a requirement catalog for a mobile application, based on current international recommendations [26]. 

A kick-off workshop with international experts was conducted to discuss the current state of medical education experience and knowledge in the international graduate medical education context. A series of publications in the Swiss Medical Journal covered various aspects of CBME and EPA development [13], [18], [20], [21], [22], [23], [24]. As a foundation for the development of specialty-specific EPAs by the professional societies, the EPA Commission developed a template (EPA-Template) based on the corresponding AMEE guide [7] and published it on the SIME website [17]. This template includes all necessary elements of an EPA with explanations and comments to assist the professional societies. Additionally, a revision process for the creation of EPA-based residency programs was developed.

### EPA development at the professional society level

As of October 2023, 28 out of 45 professional societies have utilized the consultation services of the EPA Commission and formally commenced the identification and development of suitable EPAs for their respective training programs or sub-specializations, see table 1 (Tab. 1) and table 2 (Tab. 2). Some professional societies have already published their preliminary results, and in the case of the cardiology society, they have aligned their EPA-based curricula at the European level [27], [28]. The EPA development processes at the professional society level vary and are adapted to the respective professional contexts. Typically, specialty-specific EPA working groups are formed by the professional societies, which are advised by the EPA Commission throughout the development process. Guidelines for EPA development have been published by the EPA commission [24].

Since the beginning of the reform process, initial pilot hospitals have been identified for the implementation trials of EPA-based training curricula. For these pilot training sites, an additional working group has been established to focus on the development of transdisciplinary EPAs (“common EPAs”) [29].

For over ten years, the SIME has maintained a close collaboration with the Royal College of Physicians in London for continuous faculty development. Each year, English-language workshops are offered for clinical supervisors (primarily senior physicians) to advance their skills in medical education [19]. This program has been adapted to the national context of Switzerland in terms of content and language, with a particular focus on competency-based medical education, including EPAs. Annually, more than 30 workshops are held across three language regions. Additionally, for young senior physicians with a special interest in education, a compact course is offered once a year (Swiss Medical Education Summer School) [25]. Approximately 30 instructors are available for the entire Teach-the-Teachers program, speaking German, French, and Italian.

## Discussion

The competency-based graduate medical education reform in the Swiss context has been successfully initiated. The foundation for this was a national strategy (“Unité de doctrine”) with a clear leadership structure [30], the establishment of a national core team, an advisory board comprising international experts, and a comprehensive faculty development program. A large proportion of professional societies have made substantial progress in the development of EPAs, a crucial element of the graduate medical education reform. Results of these efforts have already been partially published [27], [28]. The upcoming phase will involve initial implementation trials in selected pilot hospitals. The first transformation cycle of this training reform will be completed with the introduction of a mobile application for the documentation of workplace-based assessments and its integration into a digital graduate medical education portfolio. The results from ongoing program evaluation will be used for the subsequent reform cycle.

A significant challenge in the context of the described graduate medical education reform is the adaptation of international medical education recommendations and standards to national and local contexts. It has already become apparent in this early phase of the reform that “thinking in professional society silos” regarding training content and structures can be an obstacle to training reforms. At the same time, it appears that competency-based graduate medical education, in addition to improved operationalization of clinical training and better supervision and feedback quality for trainees, may also create innovative opportunities for inter- and transprofessional training [31]. Ongoing program evaluation will determine whether and how these goals are achieved.

## Conclusion

The competency-based transformation of graduate medical education with the introduction of EPAs is a multilayered change process. Experience so far indicates that continuous exchange and coordination among stakeholders are necessary to develop a shared mental model of competency-based training. If this is achieved, a genuine improvement in graduate medical education and, consequently, in patient care, appears possible.

## Authors’ ORCIDs

- Severin Pinilla: [0000-0002-0797-2043]
- Jan Breckwoldt: [0000-0003-1716-1970]
- Christoph S. Burkhart: [0000-0002-9288-117X]
- Eva K. Hennel: [0000-0002-7625-5785]
- Adrian P. Marty: [0000-0003-3452-9730]
- Urs von Wartburg: [0009-0001-5239-4174]
- Monika Brodmann Maeder: [0000-0001-5608-7887]
- Sören Huwendiek: [0000-0001-6116-9633]

## Acknowledgements

We would like to extend our gratitude to the other members of the EPA Commission: Sonia Frick, Pierre-André Michaud, Gloria Bünzli, Sarah El Hamouri, Ulrich Woermann, Babara Rohr, Jérémy Glasner, Mareike Cordes, Anja Kéry-Candela and Matthias Widmer for their dedicated work. Additionally, we wish to pay special tribute to our late co-author Werner Bauer, whose optimistic, tireless, and visionary efforts made the advancement of graduate medical education in Switzerland possible.

## Competing interests

The authors declare that they have no competing interests. 

## Figures and Tables

**Table 1:**
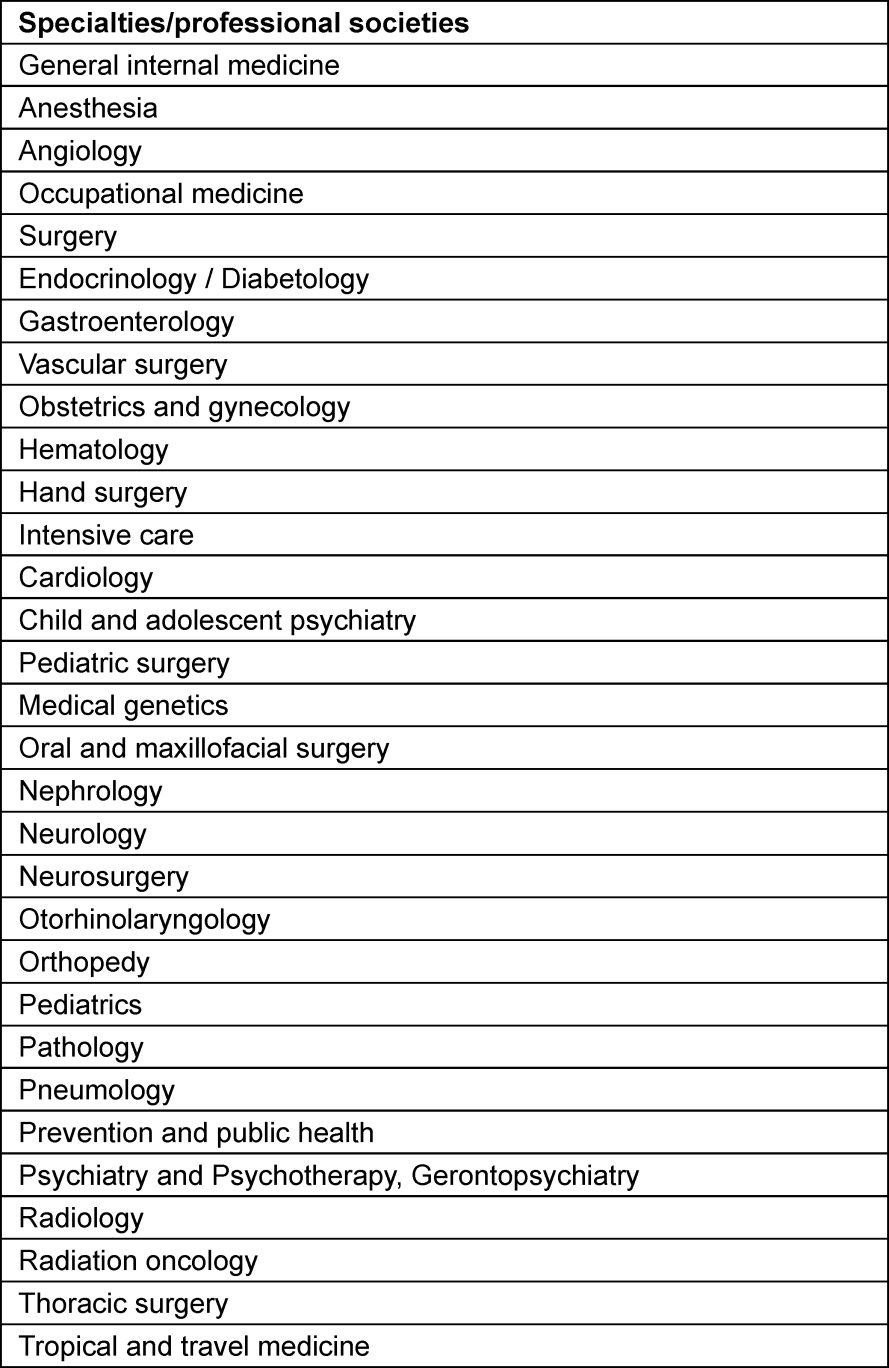
List of specialties/professional societies that are already developing and/or using EPAs. This is an ongoing process, and the table reflects the status as of October 2024.

**Table 2:**
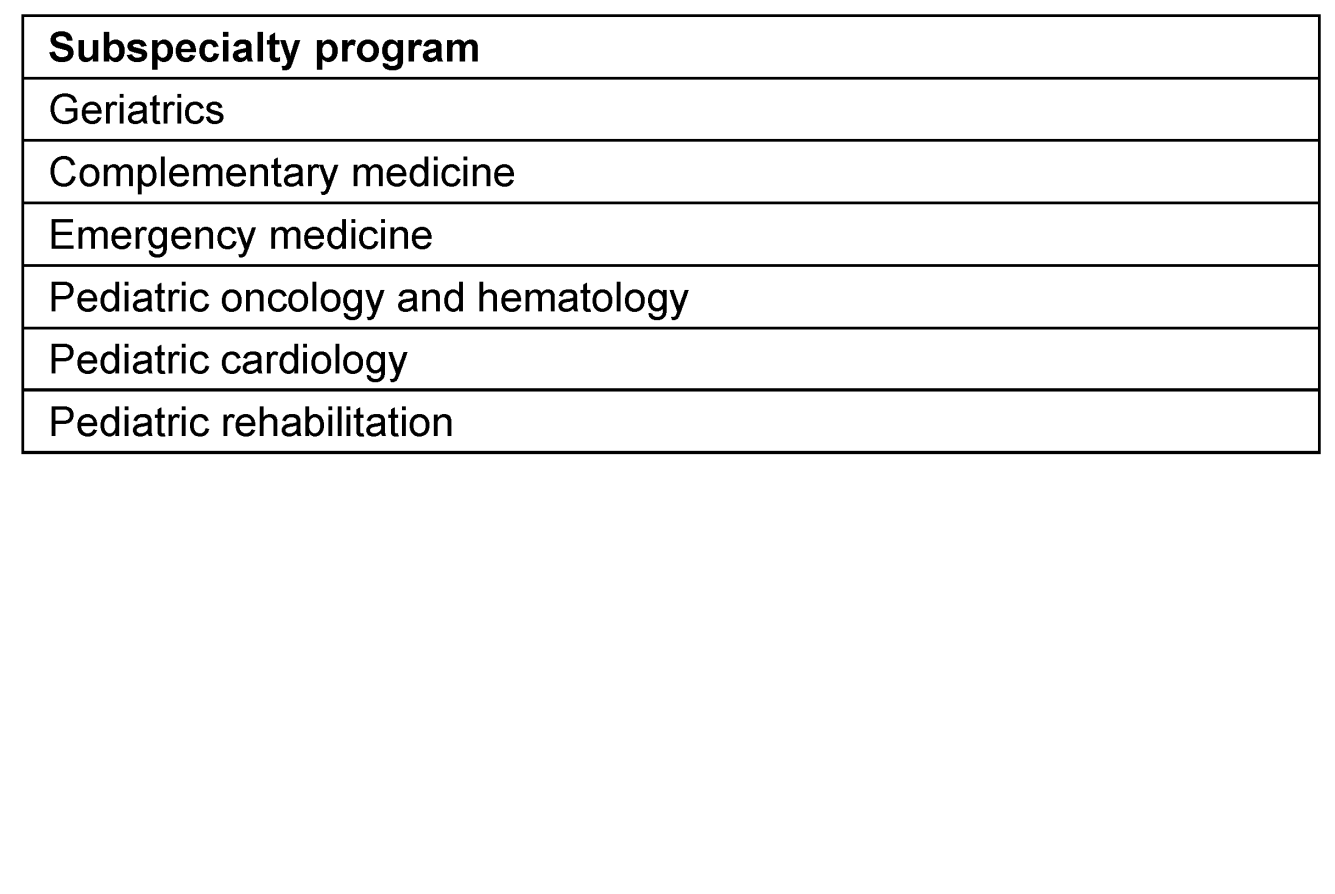
List of subspecialty programs for which EPAs are already being developed. This is an ongoing process, and the table reflects the status as of October 2024.
